# Safety and Reverence: How Roman Catholic Liturgy Can Respond to the COVID-19 Pandemic

**DOI:** 10.1007/s10943-021-01282-x

**Published:** 2021-05-24

**Authors:** Sergey Budaev

**Affiliations:** Department of Biological Sciences, University of Bergen, Postboks 7803, 5020 Bergen, Norway

**Keywords:** COVID-19, SARS-CoV-2, Airborne transmission, Sacraments, Mass

## Abstract

The current COVID-19 pandemic is a major challenge for many religious denominations. The Roman Catholic Church strongly depends on physical communal worship and sacraments. Disagreements grow concerning the best balance between safety and piety. To address this issue, I review the major transmission risks for the SARS-CoV-2 virus and list certain measures to enhance the safety of the Roman Catholic Liturgy without compromising its intrinsic beauty and reverent spiritual attitude. This can be achieved through assimilation of several traditional elements into the modern liturgy. I emphasize that religious leadership and decision-making should be transparent and based on inclusiveness, pluralism, best scientific evidence and voluntary cooperation.

## Introduction

Epidemics have long accompanied the human history (Hays, 2009). Within the last hundred years, we have experienced a deadly Spanish flu pandemic with caused from 50 to 100 million deaths (Nickol & Kindrachuk, 2019). Just a decade ago, we came through the H1N1 swine flu influenza with the global mortality burden 151,700–575,400 (Dawood et al., 2012). Now society is challenged with the COVID-19 pandemic: lives lost, health systems overloaded and national economies devastated. People worldwide are increasingly tired of fear, lockdowns and restrictions. All this adversely affects the mental health of the world population, which further aggravates the health damages through increased morbidity (Torales et al., 2020).

In the past, the Church^1^ tended to be on the frontline, establishing hospitals, caring and providing for the sick and poor, as well as delivering the necessary spiritual help (Bassareo et al., 2020). The historical Catholic Church supported quarantine, lockdowns and church closures (Arrigoni, 2020). Catholic hagiography provides examples of responsible social distancing during earlier pandemic (Perciaccante et al., 2021). However, with only rudimentary development of the evidence-based scientific method, minimal empirical knowledge and absence of an efficient health care system, it was difficult to find the optimal balance between the spiritual, compassionate, health care and epidemiological objectives. There were clashes between public worship and government quarantine regulations even in more recent history.

We keep the Christian hope and scientific aspiration that every pandemic will find its end. The current COVID-19 situation has a tendency towards improvement, thanks to the unprecedented public health measures and development of efficient vaccines. However, we live in an increasingly globalized world, confronting global environmental problems and climate change. This unfortunately means that humanity may encounter similar challenges in the future. It is important to take lessons, not only epidemiological but also societal and spiritual. What are the public health implications of various religious practices and rituals? How can they be adapted to minimize the spread of airborne infection but still not compromise their intrinsic spiritual value and beauty? Which adaptations introduced during the COVID-19 crisis could be recommended long-term?

An initially overcareful response to the pandemic could have been justified since the precautionary principle (Martuzzi & Tickner, 2004) can be applied given huge uncertainty. Public decision-making was based on misinformed models in the absence of good quality data (Ioannidis et al., 2020; Joffe, 2021). Growing scientific evidence, improvement of mitigation measures and the arrival of efficient vaccines now allow for better, more balanced decisions. In this work, I try to provide a brief outline of the major epidemiological risks associated with the COVID-19 pandemic. I also propose certain measures to enhance the safety of the Roman Catholic Liturgy without compromising its intrinsic beauty and healthy reverent spiritual attitude. There should, indeed, be no inherent conflict between the work of God and the safety of co-workers in God’s vineyard.

## Challenges for Church and Society

In-person public gathering is one of the most essential components of worship in all Christian denominations as well as in most non-Christian religions. During the COVID-19 pandemic, research has shown that mass religious events linked to worship and holidays played an important role in the initial spread of the SARS-CoV-2 infection (Aherfi et al., 2020; James et al., 2020; Pung et al., 2020; Vermeer & Kregting, 2020; Yezli & Khan, 2020). Certain faith-based communities resisted the efforts of the state agencies to limit religious gatherings (Hill et al., 2020). When the health authorities introduced social distancing and restricted mass gatherings, some communities continued worship (Capponi, 2020; Levin, 2020; Singh, 2020; Wildman et al., 2020). Certain groups even went into direct conflict with the civil authorities suing them for violation of the religious liberty rights (Corbin, 2020). It can be accepted that the primary motivation for even the most rebellious groups is the Christian Love with the willingness to help, care and take the heavy burden, as well as the Christian call to not be in fear (Levin, 2020). However, the outside uninformed society sees selfishness and self-focus with little practical help to diminish the damage. This may create a distorted image of Christianity as a rigid cult, associated with egoistic tendency to keep favoured rituals at any cost to the society. Arguably, this could place under indiscriminate fire even those communities that try to be loyal to the health care regulations (Just, 2020).

Thus, epidemiological regulations to mitigate the spread of COVID-19 can come into conflict with the faith values and practices (VanderWeele, 2020). For example, sacraments, especially the Penance and the Eucharist, are indispensable for any faithful Catholic. Being deprived from them—even lawfully—is for many extremely distressing emotionally, creates a strong sense of loss, and could lead to anxiety, depression, anger etc., negatively affecting both physical and mental health (VanderWeele, 2020). Further, even relatively small changes in the ritual and religious practice explained by the need to mitigate further spread of the infection can be perceived by some faithful as unacceptable degradation of the religious piety, insulting abuses that are detrimental to the eternal life and the true standing of the Universal Church. This could cause tensions within the community, bringing overinterpretation, rumours, stigmatization and marginalization. Further, this could create suspicion to the religious leaders and even give rise to conspiracy theories (e.g. Kokx, 2020). During the General Audience 6 September 2020, Pope Francis acknowledged this inherent problem by stating that “*gossip closes the heart of the community, closes the unity of the Church*” and “*Gossip is a plague worse than COVID.*”

The pandemic continues to create tensions within the religious communities. The author joins the regret that Catholics often disagree on so many matters. “*One might, however, have hoped that all Catholics would by now have substantially agreed with each other when it comes to dealing with the Covid-19 virus. But they have not.*” (Davies, 2020, p. 503). This is unfortunate because in the past, changes in the religious practices caused painful separations and even permanent schisms.

It can be argued that adapting the religious life to the pandemic era includes many sensitive issues that cannot be efficiently resolved through direct top-down administrative enforcement. A significant degree of voluntary cooperation, transparency and trust is required for all parties: the faithful, religious leaders, state authorities, physicians and scientists. Furthermore, the solutions should be based on the best available scientific evidence, religious prudence and allow for a degree of tolerance, inclusiveness and pluralism, given the diversity of most typical church communities. This is especially true for the Catholic Church, which often represents a diverse cultural mix with a large proportion of people with immigrant background. The ultimate means for such adaptation is the Christian Love, compassion, cooperation and solidarity (Levin, 2020; Serving a wounded world…, 2020). Religion is one of the most important, intimate, parts of life for many citizens. The authorities must therefore express understanding and flexibility to keep trust, unity, solidarity and collective resilience. Stronger effort promoting prosocial attitudes are needed to help overcome negative effects of COVID-19 pandemic and other disasters (Politi et al., 2021).

Recent research on leadership and governance agree that the most efficient rapid response to the COVID-19 pandemic requires inclusivity, transparency and participation of wider society in the decision-making process at all stages. Decision-making should also place much effort on communicating accurate evidence-based scientific information for risk assessment and management, accounting also for the uncertainty and multiple side effects (Moon, 2020; Rajan et al., 2020). The principal importance of transparency, accountability, informational accuracy and inclusivity for efficient response to the challenges posed by the current pandemics to faith-based communities has been emphasized (Levin, 2020). In contrast, public health measures not based on the best scientific evidence, inaccurate, or exaggerated information could bring harm (Ioannidis, 2020; Pearce et al., 2020). This is especially important because framing the pandemic in global media often lacked coherence and tends to facilitate fearmongering due to obsession with breaking news (Joffe, 2021; Ogbodo et al., 2020). All this also significantly contributed to an infodemics of rumours, false information, and conspiracy theories (Islam et al., 2020; Levin, 2020; Pennycook et al., 2020) damaging both Christianity and the wider society. As a significant collateral harm, this also would undermine the public trust in science that has been building up for the last century but has more recently been slowly eroding (Resnik, 2011). Yet, it is known that trust in science is one of major predictors of compliance with the health care measures (Plohl & Musil, 2020), so this would be detrimental for the public health in the end. What we urgently need is to develop the synergy between the science, civil authorities and faith (Hong & Handal, 2020; Levin, 2020).

## Risk Factors for COVID-19 Transmission

Current scientific evidence indicates that COVID-19 is mainly transmitted via small respiratory droplets during close face-to face contact (Wiersinga et al., 2020) and airborne transmission via exhaled aerosol (Chen et al., 2020; Morawska & Cao, 2020; Scheuch, 2020; Stadnytskyi et al., 2020). Coronavirus transmission with food (Eslami & Jalili, 2020) and via fomites on inanimate surfaces are not a significant risk factor in community situations (CDC, 2021; Colaneri et al., 2020; Goldman, 2020). Asymptomatic transmission the of virus is also uncommon, although presymptomatic carriers account for a significant proportion of all transmission (Wiersinga et al., 2020). For children under 10, the risk, incidence, and severity of the disease are much smaller than in adults (Boast, 2020; Choi et al., 2020) with minor transmission risk (Boast, 2020; Danis et al., 2020; Park et al., 2020). Context-specificity, stochastic factors and individual level variation play a major role in the global SARS-CoV-2 spread, emphasizing strategies focused on avoiding superspreading (Althouse et al., 2020; Endo et al., 2020; Fang et al., 2020; Sneppen et al., 2021; Van Damme et al., 2020) rather than blanket lockdowns (Bendavid et al., 2021; Joffe, 2021).

Various respiratory activities, such as breathing, talking, coughing and sneezing, cause droplets of diverse sizes composed of saliva or mucous coating of the lungs that are emitted and carried by the air burst (Seminara et al., 2020). Larger droplets travel ballistically through the air and fall down via the force of the gravity. Smaller droplets and microscopic dried-out droplet nuclei form a cloud that could stay in a suspended state for longer time (Chen, 2021; Scharfman et al., 2016; Tang et al., 2006). Very small aerosol particles could remain in the air very long and potentially spread to larger distances: such airborne transmission can provide a major risk factor for COVID-19 transmission (Chen et al., 2020; Scheuch, 2020; Seminara et al., 2020; Zhang et al., 2020).

Airborne particles could be carried with (and interact with) various indoor air movements, such as the thermal plume: convection flow induced by heaters and the human body temperature exceeding that in the environment. Such thermal plume has various complex effects and could produce vertical air velocities comparable to an average wind speed in an indoor environment (e.g. Li et al., 2013). In some circumstances, thermal plume could transfer the suspended particles to the breathing zone and contribute to infection spread (Salmanzadeh et al., 2012). In some cases, thermal plume can make an air curtain protecting from the airflow emitted by the others (see refs in Wei & Li, 2016). Additionally, at low air mixing, thermal plume can efficiently raise contaminated air upwards from the breathing area (Mui et al., 2009; Vuorinen et al., 2020). However, thermal stratification in the room can cause the exalted air to lock at a certain height, including the breathing level (Liu et al., 2020). Forced air movement could spread contaminated exalted air to significant distances. For example, air recirculation mixing is likely to cause significant spatial spread of contaminated aerosol (Mui et al., 2009) and increase the virus transmission risk (Wei & Li, 2016). It has long been known that deficient indoor ventilation helps spread airborne infections (Li et al., 2007). Even human walking can induce significant amount of air flow, especially if it is sustained (Bhattacharya et al., 2020). Provided the SARS-CoV-2 virus is transmitted via aerosol and fine droplets, the exposure time becomes the major factor which should be controlled in addition to the distance (Vuorinen et al., 2020).

It is well known that the size distribution, quantity of droplets, their emission rate and distance significantly increase from breathing to speaking, coughing and sneezing (Chao et al., 2009; Schijven et al., 2020; Seminara et al., 2020). Normal breathing usually emits a small amount of particles with low peak speed, resulting in short distance transport (Wei & Li, 2016; Zhao et al., 2005). Nose-only breathing has an even more limited potential to transmit airborne infection than mouth-breathing (Bazant & Bush, 2021; Xu et al., 2017), especially if the exhalation vector is angled downwards (Renzi & Clarke, 2020). Loudness of speaking correlates with the rate of droplet transmission (Asadi et al., 2019) and phonetic characteristics strongly affect the air transport (Abkarian et al., 2020). Droplet production while speaking could approach that while coughing (Chao et al., 2009; Schijven et al., 2020). Normal speech has been linked with airborne transmission of SARS-CoV-2 virus (Stadnytskyi et al., 2020). Furthermore, superspreading events are directly linked to the wide transmission of airborne aerosol as a consequence of choir singing in a confined indoor environment (Miller et al., ﻿^2021^; O’Keeffe, 2020).

It has long been known that ventilation is essential to avoid airborne infections. However, it must involve intake of uncontaminated air from the outside: mixing-only ventilation would, on the contrary, increase the risk of airborne infection (Li et al., 2007). This is correct also for the SARS-CoV-2 virus (Chen, 2021; Dai & Zhao, 2020; Morawska et al., 2020). Intake of fresh air from outside plays in concert with the social distancing and reduction of the occupant density (Jones et al., 2020; Sun & Zhai, 2020). Even mild ventilation can effectively reduce infection risk under low viral load (Liao et al., 2005). However, ventilation-induced airflows can interact with the thermal stratification and human- and heater-induced thermal plume. For example, downward ventilation (with the exhaust at the bottom) could compromise the upward air transfer precluding effective removal of the contaminated air (Wei & Li, 2016). Other studies confirm that ceiling-level ventilation exhaust is more efficient in removing exhaled particles (Qian & Li, 2010).

Personal protection, such as surgical face masks, has been recommended for mitigation of COVID-19 transmission, especially for the source control. While the N95 respirators provide a sufficient protection against exhalation aerosol, cheaper and more common surgical and cloth mask give only about 30% protection of N95 (Bowen, 2010). Theoretical calculations are more optimistic (Dai & Zhao, 2020). Surgical masks reduce the dispersion of exalted air (Hui et al., 2012) and can diminish the amount of influenza and coronavirus RNA in the exalted air (Leung et al., 2020). Wearing a mask, therefore, can efficiently prevent SARS-CoV-2 virus transmission from symptomatic and presymptomatic carriers. Recent studies suggests that face masks, in addition to the standard social distancing, provide significant protection if they are worn by a majority of the population (Chu et al., 2020; Howard et al., 2021; Kähler & Hain, 2020; Salter, 2020). It should be stressed, nonetheless, that the evidence for the protective effect of face covering alone, while compelling, remains relatively low (Chou et al., 2020, 2021; The Royal Society, 2020). Mask efficiency could differ depending on the material, environmental conditions, fit, respiration pattern etc. (Brooks et al., 2021; Konda et al., 2020; Leith et al., 2021; Tcharkhtchi et al., 2021; Zangmeister et al., 2020). The consensus is that face covering is not a panacea but compliments social distancing and other public health measures. The WHO advises wearing face mask by the general public, but stresses that its applicability depends on combination with other measures and should be based on risk analysis in each case (WHO, 2020a).

The significant role of the smallest droplets and fine aerosol in the transmission of SARS-CoV-2 virus makes the conventional social distancing rule, 1–2 m, rather simplistic (Bazant & Bush, 2021). The level of risk, instead, should take into account the kind of the environment, duration of the indoor exposure, characteristics of the ventilation air exchange, the intensity of the indoor air flows, the kinds of the occupants activities indoor (e.g. physical exercises with intense breathing, singing, talking or being quiet), possible use of personal protective measures (e.g. face masks) and other factors (Dbouk & Drikakis, 2020; Jones et al., 2020; Morawska & Cao, 2020; Sun & Zhai, 2020). For example, a temporary exposure in a well-ventilated room with dispersed occupancy and minimal speaking activity (silent) is safe in absence of personal protection, whereas speaking raises the infection risk to the medium level (Bazant & Bush, 2021; Jones et al., 2020). In this way, temporary exposure in a well-ventilated environment without personal protection and no speaking activity can make for a medium risk even at high occupancy (Bazant & Bush, 2021; Jones et al., 2020).

## Balancing Risk Assumptions

The Roman Catholic Mass (as well as other worship services) normally involves one or several priests and lay congregation.^2^ There can be one or several altar servers assisting at the Mass, who are typically also lay members. There are often one or several extraordinary lay ministers who help the priest to distribute the Eucharist to the congregation.

The priest is the key element of the Roman Catholic Mass: it cannot occur without him (*Codex juris canonici*, canon 900), but can be celebrated unassisted by servers and does not necessarily require extraordinary ministers. The celebrant priest cannot be silent while saying the Mass. It is also the priest who necessarily distributes the Holy Communion to the faithful. This makes him the main potential “single point of failure” (using the technical concept, e.g. see Lynch, 2009): If the priest celebrating the Mass happens to be infected presymptomatically (or with symptoms that of course should be avoided by all means), this would place anyone in the congregation at risk. Protecting the celebrant from the infection is, therefore, the highest priority.

The numerous congregation members are much more difficult to control than a single priest, they have much more degrees of freedom. People could have many close contacts, permanent and transient: family, job, friends, school/university, neighbours, strangers in public transport, at shopping etc. This would increase their chance of catching infection and therefore bring it presymptomatically to the Mass. Note that the probability of catching an infection in potentially numerous independent and mutually exclusive situations (in *i*-th case having individual probability *p*_*i*_) ⋃*p*_*i*_ is equal to the sum of their individual probabilities Σ *p*_*i*_.

This might well be true also for the priest. However, it would seem easier to limit the size and diversity of the social network of close contacts for the priest than for any random member of the laity. Indirect data suggest that the challenging demands of the priestly vocation in the Roman Catholic Church brings about a reduced network of close relations and support (Bricker & Fleischer, 1993). Finally, the fact of numerosity of the congregation members will inflate the statistical probability for an infected person to occur among them by chance alone. Assuming the infection probability is *p* and there are *n* statistically independent participants, the probability that at least one of them is infected would be *1-(1-p)*^*n*^, i.e. raises exponentially with the group size, quickly approaching 1 (﻿see Brown & Mangel, 2021). For a large gathering, there can be a high probability that at least one participant is infected even if the infection risk *p* is low overall. Thus, it can be assumed that the risk of being infected is normally smaller for the priest than for the congregation.

## Mitigating Transmission Risk During the Liturgy

All the above discussion suggest the following measures to mitigate the risk of airborne transmission of SARS-CoV-2 virus at the public Mass. I will loosely follow the “hierarchy of control” concept for the levels of hazard management (CDC, 2020b) involving increasingly less efficient measures: physical elimination of the hazard, isolating people from the hazard, administrative measures to avoid hazard and finally individual protection. Full elimination of the hazard is impossible because of the Catholic tradition principally depends on the physically administered Sacraments, especially Penance and the Eucharist. Unlike certain other denominations, “virtual communion” (e.g. Evener, 2020) is not a solution for Catholics, even though there is a practice of Spiritual Communion. The considerations below agree with the general recommendations of the World Health Organization (WHO, 2020b, c) and Centers for Disease Control and Prevention (CDC, 2020a) but are adapted specifically for the Roman Catholic liturgical tradition. Some measures, such as social distancing and frequent cleaning, have already been widely implemented in various parishes worldwide.

### Social Distancing

First and foremost: anyone having respiratory symptoms should not show up at any public worship. All members of the congregation should observe the social distancing and avoid to come in direct contact with one another before, during and after the Mass. This concerns not only friendly hugs and handshaking, but also talking. If the church has several porches, it can be good to separate the entrance and the exit, so that all visitors go in approximately one direction and not mix. This would help avoid congestion and minimize face-to-face contacts before and after the service, when the participant traffic is high. The arrows pointing the direction of the traffic can be marked on the floor, together with marks indicating the minimum distancing. There should also be separation in the pew use, so that all participants could sit apart in a chess order, ideally with every other pew blocked out of use. Seats can be clearly marked to assist the congregation members in keeping the chess order and the appropriate distance. The congregation should avoid unnecessary walking during the Mass that causing extra air transport around the indoor environment (this is rarely an issue). The Sign of Peace should not involve handshaking or any other close contact, including vocal greeting: just a sincere, silent bow is enough. The priest should not roam in the nave to avert possible exposure to contaminated air. To minimize fomite-based risks, surface cleaning should be implemented and several hand sanitizers should also be provided.

### Ventilation

It is crucial to avoid accumulation of and exhaled air that can contain infectious aerosol. Therefore, good ventilation is important. However, many historical churches do not feature efficient mechanic ventilation systems or air conditioning with outdoor intake. Passive air infiltration is usually inefficient. Nonetheless, intentional airing through open doors and windows would provide good results (Hayati, 2018). For example, a single-sided airing for one hour ensures approximately 50% air exchange even if it is conducted through one small side door (Hayati et al., 2017). It is therefore recommended to air the church building for a comparable time (depending on the number and size of the doors, windows and practicality of their opening) before and after the public worship. However, the use of fans should be avoided during the service because their air flows can spread contaminated aerosol very far from the source. If rapid heating is needed in the cold climate, it can be done using a fan heater, but only before the gathering. When other mitigation measures are performed, it would be safer to allow the relatively small amount of possibly contaminated air to localize near its source during the worship and then dilute and remove it afterwards through airing.

### Singing and Other Vocal Activity

The congregation will significantly reduce the risk of spreading infection through reduction of their vocal activity, even though this might interfere with the *active participation* in the Liturgy. In particular, loud communal choral singing should be avoided. Liturgical verbal responses of the faithful can better be said in low voice. However, singing could be delegated to a choir, separated from the other parishioners. There should ideally be few choir members to reduce the production of aerosol potentially contaminated with virus particles and diminish the statistical probability that someone is infected by chance (see above). Many historical churches and cathedrals have designated choir or quire area. To further protect the celebrant from the infection risk, common placement of both the clergy and the choir, such as in the quire area, should be avoided. Many churches feature the choir at an elevated platform. A displacement ventilation system with top exhaust could be installed in such an arrangement. This would ensure both sufficient intake of fresh air to the choir area, its fast removal, along with reduced air mixing and disturbance in the main nave. As the other participants of the Mass, the choir should observe social distancing. Further, to block wider transfer of aerosol associated with the singing, plexiglass shields can be fitted. These can be removable to avoid changing the historical arrangement of the interior. More specific advises for organizing singing and music during the pandemic could be found in specialist publications (Naunheim et al., 2020).

### Active Participation in the Liturgy

The Constitution of the Second Vatican Council *Sacrosanctum concilium* states that “*all the faithful should be led to that fully conscious, and active participation in liturgical celebrations which is demanded by the very nature of the liturgy. Such participation by the Christian people as "a chosen race, a royal priesthood, a holy nation, a redeemed people (1 Pet. 2:9; cf. 2:4–5), is their right and duty by reason of their baptism*” (14). However, there is no indication that playing a part in singing is mandatory for the faithful. Active participation means full conscious attention, which can involve an internal, spiritual rather than ostensible vocal, verbal or other bodily dimensions. As Pope Benedict XVI wrote in “*The spirit of the liturgy”* (Ratzinger, 2000), “*The real liturgical action, the true liturgical act, is the oratio, the great prayer that forms the core of the Eucharistic celebration …*” (p. 172) and “*We should be clearly aware that the external actions are quite secondary here. Doing really must stop when we come to the heart of the matter: the oratio.*” (p. 174). Abstaining from singing can therefore be a conscious act of humility and compassion that stems from solidarity with those who have been suffering so much loses from the pandemic.

### Concelebration, Altar Servers and Extraordinary Ministers

The Church Law currently allows and even encourages the priests to concelebrate the Eucharist to express the *unity of priesthood* (*Sacrosanctum concilium* 57*;* CJC, canon 902; Guidelines for concelebration of the Eucharist, 2013). However, this common practice is not automatic and depends on the “*welfare of the Christian faithful*” (CJC, canon 902). The current COVID-19 pandemic situation may require to limit this because it may compromise social distancing among the priests. As the above discussion points, the celebrant requires the maximum protection. However, as long as concelebrating priests are well known to each other and frequently interact otherwise, this limitation can be relaxed. Limiting concelebration would not in any way compromise piety. On the contrary, the ages-long practice of the Latin Rite before the Second Vatican Council did not allow it. More traditionally minded Catholics point that it could obscure the unique role of Jesus Christ and clericalize the Liturgy. A call to limit concelebration to more extraordinary, solemn occasions is quite common, especially because it has in some occasions been misused (e.g. Kerr, 2012). Altar servers significantly interact with the Celebrant during the Mass, making strict social distancing hardly possible. Thus, as long as it is difficult to always guarantee their good health status and ensure distancing, their number should be minimized, in many cases to just one person well familiar with the Celebrant. There is no limitation for celebration of the Holy Mass unassisted. The Extraordinary ministers, helping distribute the Holy Eucharist among the faithful, represent a high-risk position because they interact closely with both the Celebrant and many faithful. They are also typically lay persons that have as many social contacts. While the priest always have good training and prolonged experience of distributing Holy Communion, Extraordinary ministers would not. Temporary suppression of their service at the Liturgy during the pandemic would minimize a potentially important infection vector. Finally, celebration of the Liturgy in smaller chapels may pose additional challenge. One protective measure is to serve facing the altar: *Ad orientem*. This not only strengthens our focus on Our Lord, has deep symbolism, but would reduce the Celebrant’s exposure to the respiratory aerosols and small droplets generated by the congregation, who most of the time remain at the back of the Celebrant.

### Personal Protection

Because the widespread use of face masks can reduce transmission risk, especially in a confined indoor space, they can be recommended. Additionally, disposable gloves have initially been suggested. However, the fomite vector later appeared of minor importance for COVID-19 and the use of gloves was criticized for compromising traditional reverence and piety to the Eucharist. The probability that the celebrant is infected is small (see “Balancing risk assumptions” section), the priest takes maximum precaution to keep hands clean as a normal element of reverent treatment of the Holy Sacrament. It is very unlikely that he touches potentially contaminated foreign objects during the Mass. Therefore, the need to wear gloves for the priest is questionable (see Goldman, 2020). The WHO recommendations for religious communities currently refer to the use of disposable gloves only in the context of safe burial practices (WHO, 2020c). On the other hand, face masks can be worn by the congregation before and after the Mass, when entering to and exiting from the church. This period is characterized by the highest risk of crowding, chances of bumping into the others and close contacts while at the porch. Walking is a mild physical activity, especially if entering the church involves going up the staircase. All this his may increase breathing intensity and aerosol production. Furthermore, intense movement by many people would increase air mixing that would raise the risk of wide transmission of the aerosol if some of the people is infected with the SARS-CoV-2 virus. The intensity of speaking is expected to be higher at this time, some parishioners may greet one another, have a few words to each other and so on, such instinctively affectionate activity being difficult to control. There would not be a strong risk if the masks are removed from the face when all participants of the Mass sit silent on their places, safely distanced. Thus, celebration of the Mass can proceed without face covering provided the other mitigation measures are observed. After the Mass is ended, participants can put the mask back on for safe exit from the church.

## Holy Communion

The Eucharist is the culmination and the most Sacred Mystery in the Liturgy of the Catholic Church. Catholics believe that the Holy Eucharist is the Body, Blood, Soul, and Divinity of Jesus Christ, really, truly, and substantially present, united in His one Divine Person (Catechism of the Catholic Church, 1993). From approximately IX century, reverently receiving the Holy Eucharist while kneeling and on the tongue has been the only allowed practice (Schneider, 2013). Instructions of the Congregation for Divine Worship *Memoriale Domini* (1969) and *Redemptionis Sacramentum* (2004) permitted—as an exception and under limited circumstances—distributing Holy Communion in the hand. Even though this practice has become widespread, the above documents state that it remains extraordinary and any faithful always has the right to receive Holy Communion on the tongue: according to the Canon Law, no priest or bishop has the authority to limit or forbid it for whatever reason (FIUV, 2020; Schneider, 2013).

In the current COVID-19 pandemic situation, some local Catholic authorities decided to limit distribution of the Holy Communion in the hand only referring to WHO recommendations (WHO, 2020c). However, the position of the WHO is wisely generic: it points to communion in the hand, but without direct endorsement. The document clearly states that “*Some religious leaders and faith communities have encouraged their members to receive a blessing from at least 1 m away and avoid the distribution of Holy Communion that involves placing the wafer on the tongue or drinking from a common cup*” (WHO, 2020c, p. 2).

Nonetheless, the limitations to receive the Communion in the normative way, on the tongue, is causing tensions within the Catholic Church, many faithful consider it stressful, unlawful abuse that degrades religious piety (Dodd, 2020; LMS, 2020; Schneider, 2020). Furthermore, the decision to limit the normative manner usually lack transparency, discussion and agreement with the whole community and is explained by vague hygiene without scientific evidence. Apparently, this blindly follows the generic recommendations without adapting them to the Roman Catholic Church.

The belief that the Sacrament of Eucharist can spread infection is far from new. There were several studies, mainly focusing on the use of common chalice, indicating that there may be some contamination, however, no evidence for transmission of any infectious disease has ever been documented (Anyfantakis, 2020; Gill, 1988; Hobbs et al., 1967; Pellerin & Edmond, 2013). For example, it was concluded that communion from a single chalice does not sufficiently increase the risk of infection transmission, provided symptomatic/sick participants abstain from partaking (Pellerin & Edmond, 2013). The Roman Rite normally administers Communion under one kind to the laity, the Holy Host. The Holy Species used in the Latin Rite is nearly dry and therefore is likely to have low adhesion of outside particles, further reducing the infectious risk. While receiving the Holy Bread, the communicant normally extends the tongue forward, requiring to hold breath for a while. This reduces possible respiratory output. The traditional manner of receiving Communion on the tongue is therefore unlikely to incur a high risk of infection transmission.

**Fig. 1:**
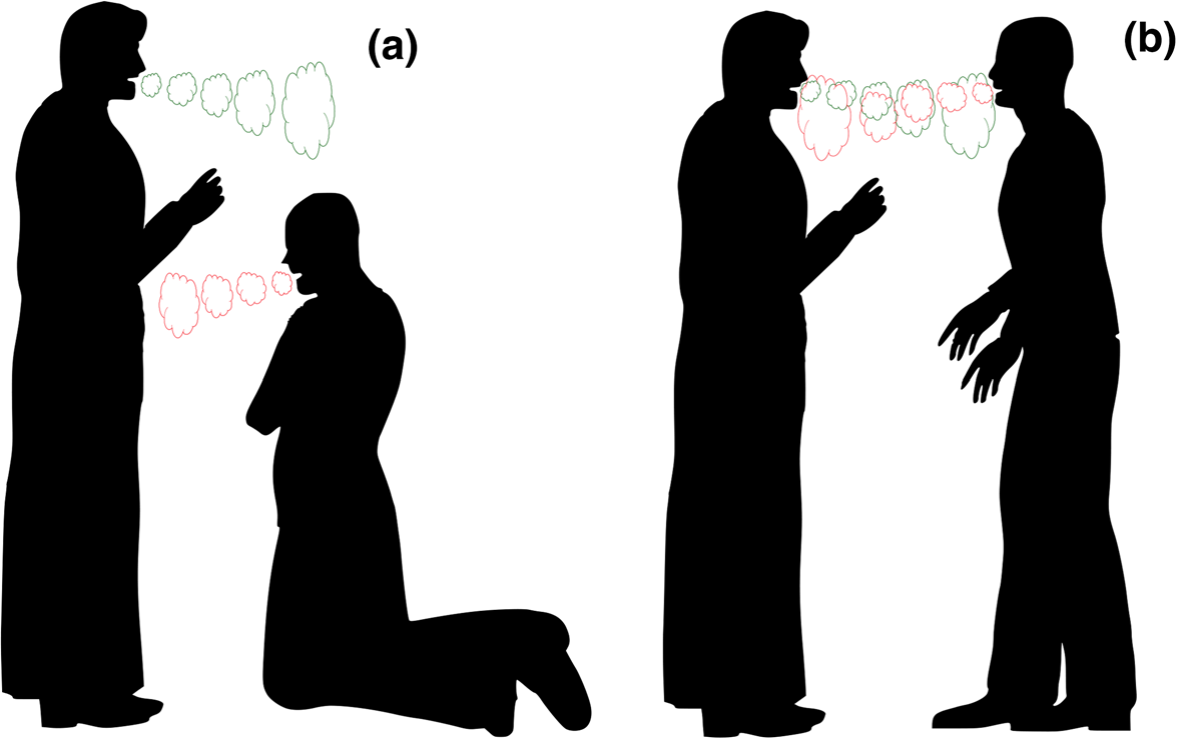
Direction of exalted aerosol while receiving the Communion kneeling (**a**) and standing (**b**). The colour of aerosol cloud depicts the level of transmission risk (green low, red high) (Color figure online)

Traditional reverent practice of the Catholic Church incorporates additional elements making it even less risky in the current COVID-19 pandemic. The kneeling position of the faithful while receiving the Host would provide spatial distancing about 50 cm (Fig. 1a): the communicant’s face is located at the level of the chest of the Eucharistic Minister. Provided the communicant stays silent, uses nasal breathing, and the duration of the interaction is short (very few seconds), this would not incur a high risk to the Eucharistic Minister (usually the priest whose safety is prioritized, see above). Furthermore, reduced verbal response of the communicant directs the droplets and aerosol towards the chest of the Minister, which is by far a lower risk than in the face.

In contrast, the typical position of the communicant for Communion in the hand is standing which is the direct, close, face-to-face interaction (Fig. 1b). Any verbal interaction between the Eucharistic Minister and the communicant would direct the droplets and aerosol directly to the Minister’s face and the Holy Bread. Inhaling such aerosol could be risky. The statistical argument (see “” above) points to an increasing probability that in a large group, at least one member is infected, further aggravating the risk to the Minister. If the communicant happens to cough or sneeze, Minister’s face and the Holy Bread become the direct target of both fine and larger ballistic droplets. This is very unlikely in the kneeling position.

Another potentially important factor is that it would be much easier for the Minister to operate fine motorics when the communicant is kneeling than standing. This is due to a much better visual feedback and more convenient hand position when the communicant is kneeling (note that fine manual workers such as watchmakers use tables for routine work rather than stands elevated to the level of their eyes). This would make it easier for the Minister to place the Holy Host optimally and safely on the tongue, avoiding contacts with the mucous membrane and the saliva.

Even though the hands of the Communicant are often assumed to be clean, there is no guarantee. Again, the statistical argument (see above) points to an exponentially growing chance for at least one person with contaminated hands to occur as the group size increases. The typical position of the hands during the prayer—directly in front of the face—makes them susceptible to contamination by exalted and ballistic droplets and aerosol. The assumption that Communion in the hand carries no or little risk is not well grounded and may in fact create a false sense of security potentially provoking more reckless behaviour of both the Minister and the communicant.

While the assumption that providing Holy Communion on the tongue is more risky than in the hand is not supported, many faithful can currently share it, especially if it have developed into a long-term habit. This would make switching to Communion on the tongue stressful for some faithful. A pluralistic approach avoiding “heavy burdens” would be better in the current situation: “See that you do not despise one of these little ones” (Mt 18:10). Thus, those who receive the Holy Eucharist in the hand may go first. This eliminates the risk that someone might fear that the Minister’s hands have been contaminated by the saliva of a previous communicant. The requirement for those receiving the Holy Sacrament on the tongue to wait their turn a the end of the queue would also be spiritually healing. First, it follows the Christian call of humility and moderation. Second, it would deter the development of false sense of superiority in those who uses the more traditional, ancient and reverent manner. We all must follow the call of Christ: “when you are invited, go and sit in the lowest place” (Lk 14:10).

Whenever possible, the Holy Communion should be received while kneeling. This is a wonderful sign of humility when we face the Greatest Mystery, it follows the ancient, reverent tradition of the Roman Catholic Church endorsed by numerous Saints and great Doctors of the Church. Additionally, it introduces vital social distancing reducing the risk of airborne transmission. It would also provide a sign of solidarity with all those who suffer various forms of isolation and rejection through the current pandemic. Some of the faithful, notably older and disabled persons, could find it difficult to kneel without support (e.g. using their hands). Then, standing position can be used. Additionally, it may be helpful to use a free-standing kneeler or use the kneeler fitted to the altar rail whenever it remains available (as in some historical churches). The manner typically used in the Extraordinary Rite—the priest approaching an already kneeling communicant—avoids face-to-face contact completely and provides further protection. The faithful who queue to receive the Sacrament should distance, avoid congestion, two-way traffic with face-to-face contact. Arrows marked on the floor could divert the traffic of those who have received the Sacrament.

### First Communion of Children

The First Communion lays down the foundation for further spiritual life, however, COVID-19 risks are much lower for children than for adults. It, therefore, must be celebrated in the most reverent and solemn normative manner. Incidentally, a similar balance of risks and benefits made leading epidemiology experts recommend early school reopening (Levinson et al., 2020) which was successfully implemented in many countries.

## Note on the Extraordinary Rite

As this analysis shows, there are several crucial components in the Roman Liturgy, which reduce the risk of airborne infection transmission: reduced verbal participation on the part of the congregation, signing by a designated choir only, no concelebration, *Ad orientem* position, no Extraordinary ministers, and receiving Holy Communion kneeling and on the tongue, with no verbal reply. Incidentally, all these elements designate the practice of the Traditional Latin Mass (the Extraordinary Rite) that has been used continuously through the ages until the Second Vatican Council. The liturgical reform of the Second Vatican Council introduced many new elements facilitating active, physical participation of the faithful in the Sacred Liturgy. However, the venerable older rite has never been abrogated: the Motu proprio by Pope Benedict XVI *Summorum pontificum* reestablished its unrestricted use in the Roman Catholic Church.

It can be argued that the long development of the old Traditional Latin Rite occurred under continuous health threats in absence of vaccination, efficient pharmacological and other technological interventions that we now take for granted. Social distancing and isolation were the only efficient strategies to mitigate wide infection spread. The Church always served the poor and the sick. This, in addition to spiritual and medical help, required solving practical epidemiological challenges. The Roman rite has developed at the centre of medieval globalization that was also a crossroad of diverse infections, and in the end largely displaced all other Latin rites. Thus, the traditional Roman Mass may not only provide a rich Christian symbolism and deep reverence to the Mystery of Faith, but also include crucial elements to mitigate—in a low-technology, non-medicinal way—a range of very contagious airborne infections: from flu to measles, chickenpox, tuberculosis and pneumonic plague. An analysis of the cultural evolution of the liturgical rites, focusing on epidemiology, would be very interesting.

## Concluding Remarks

The challenges of the COVID-19 epidemic raises both scientific and religious issues. These can be solved through transparency, inclusion and pluralism. It is suggested that the local Ordinary would establish a panel of advisors to investigate the best measures, taking account of the local circumstances and risk. This should include priests, religious, medical experts, scientists and representatives of the faithful. The substantiation for major decisions should be published in an openly available document and briefly explained in a sermon. The necessary changes would be accepted easier if they are based on rational arguments and best scientific evidence. This will also assist communication with the secular health authorities, who may look at faith-based communities with suspicion and sometimes treat the spiritual needs of citizens as “non-essential”. We are now slowly moving out from the COVID-19 despair, but the vaccination progress in many regions remains slow and significant health risks continue to persist (WHO, 2021). Several elements of the venerable old Mass can therefore be assimilated into the ordinary rite. These could not only offer better epidemiological resilience, but also promote religious piety and reverence that we need so much to struggle with challenges imposed by this and other health threats. We must remember that airborne infections threatening us include not only SARS-CoV-2: the seasonal influenza takes about 400,000 lives annually (Cozza et al., 2020; Paget et al., 2019). Therefore, traditional elements of the Latin Rite facilitating both spirituality and public health could remain in place or enabled during high-risk periods even after the coronavirus crisis is over. These could continue to help us just as these helped the previous generations of Catholics.

## Footnotes

1 This paper has a major focus on the Roman Catholic Church, but similar issues and solutions can be applicable in other Christian denominations and other religious traditions.

2 For obvious reasons, I exclude from this analysis *sine populo* Mass and private prayers that involve one person only. I also exclude any worship conducted for and within a tight and isolated religious community (e.g. a monastic), that equivalent to a single family with similar structure of risks.
